# Interactions of ions and odorant molecules with graphene-based nanostructures in synthetic urine: a molecular dynamics exploration

**DOI:** 10.1039/d5ra06390f

**Published:** 2025-11-07

**Authors:** Prasad Rama, Isabelle Simonsson, Zareen Abbas

**Affiliations:** a Department of Chemistry and Molecular Biology, University of Gothenburg Gothenburg SE-41390 Sweden zareen.abbas@gu.se +0046-766229015

## Abstract

Odor control is a burning question in many practical applications, especially in hygiene products. At present, there is a lack of understanding of how odorant molecules interact or adsorb on solid surfaces in complex solutions like urine. In this work, we have used Molecular Dynamics (MD) simulations to investigate the interaction of the odorant molecule, p-cresol at concentrations relevant to human urine (500–100 pm) on carbon surfaces. The carbon surface was modelled simply as a graphene sheet. Moreover, the graphene sheet was edge functionalized with carboxylic groups, hydroxylic and epoxy groups on its basal plane to mimic the surface chemistry of oxidized active carbon. Charged graphene surfaces were created by deprotonating either 50% or 100% of functional groups, corresponding to experimentally determined charge at various pH values. The MD simulation results showed that PO_4_^3−^, SO_4_^2−^, Ca^2+^, and Na^+^ ions form strong clusters in synthetic urine in the presence of neutral as well as charged graphene surfaces. However, ions present in the synthetic urine showed specific affinities for charged functional groups. For example, NH_4_^+^, K^+^, and Ca^2+^ showed specific affinities for hydroxyl groups, whereas Na^+^ and Mg^2+^ were complexed with both carboxylic and hydroxyl groups. Charge density and hydration have significant affect on the affinity of divalent ions. At 50% charged surface Ca^2+^ interact strongly than Mg^2+^, however at 100% charged surface Mg^2+^ showed stronger affinity than Ca^2+^ ions. The MD results also revealed that for effective adsorption of p-cresol, the hydrophobic surface such as pristine graphene is the best candidate because charging of the surface weakened the p-cresol interaction due to counterions accumulation near the charged groups, which pushed away the neutral p-cresol molecules. A general conclusion which can be drawn from the results of MD simulations is that p-cresol interaction with graphene surfaces becomes weaker in synthetic urine compared to pure water. To the best of our knowledge, this is the first study on such a system and shall trigger further work in this direction.

## Abstract 2

Interaction of odorant molecules: p-cresol (PCRO) with neutral (GRA) and charged graphene (GRO) interfaces in the complex environment of synthetic urine.

## Graphical abstract

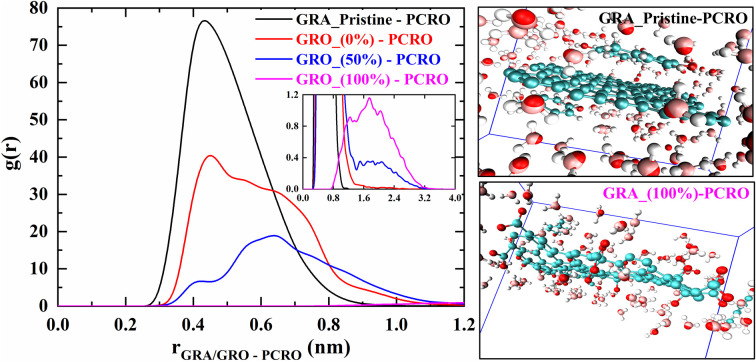

## 1. Introduction

Odor from used urinary incontinence products is a great inconvenience and has a huge negative impact on the quality of life of those affected, approximately 400 million people world-wide.^1^ The odor stems from the urine itself and odorant-producing bacteria. The invention of functional materials which can effectively control the odor are highly desirable and is an active research area. Generally, porous materials with high surface area, such as active carbon or metal organic frameworks (MOFs), are suitable for odor control.^2^ However, the effectiveness of these materials in odor control is highly dependent on the nature of the odorant molecules and interaction with the solid surface in the relevant complex solution mixture, such as urine. Urine is a complex mixture of dissolved ions, proteins, and other organic molecules. The ionic composition of human urine can vary significantly, depending on the individual's food and metabolism. Therefore, it is a common practice to generate synthetic urine (SU) in the laboratory for the investigation of processes occurring in solution or at the solid–solution interface. Over the years, many protocols for SU have been proposed and recently an updated protocol to produce a SU has been given in the literature.^3^ The major constituents of SU on which MD simulations are based are given in Table 1.

**Table 1:** Composition of the synthetic urine as obtained from ref. 3

Compound	Concentration (mmol L^−1^)
pH	6.0
Urea (CH_4_N_2_O)	250
Sodium (Na^+^)	93
Potassium (K^+^)	32
Ammonium (NH_4_^+^)	24
Calcium (Ca^2+^)	1.7
Magnesium (Mg^2+^)	4.4
Chloride (Cl^−^)	88
Sulphate (SO_4_^2−^)	18
Phosphate (PO_4_^3−^)	24

As evident, the SU is a complex mixture of salts along with urea. The urea plays a significant role in the denaturation of proteins,^4^ and therefore the effect of urea on the properties of water, as well as on the salt solution, has been investigated both by experimental methods^5,6^ as well as by simulations.^7,8^ There have been many studies reported in the literature where the thermodynamic properties of urea in different salt solutions have been investigated. Recently, Sadeghi *et al.*^9^ have shown that ion-specific effects are exhibited in the experimentally determined osmotic coefficients of urea and alkali metal chloride solutions. It has been demonstrated that osmotic coefficients of urea in salt solutions follow the order Li^+^ > Na^+^ > K^+^, whereas osmotic coefficients of urea in sodium salts with different anions follow the order NO_3_^−^ < Cl^−^ < Br^−^. The order of cations can easily be related to the hydration of ions, *i.e.*, strongly hydrated cations such as Li^+^ generates higher osmotic coefficients than weekly hydrated ions, *i.e.*, K^+^. However, the trend in anions is more complex and difficult to rationalize by the argument of ion hydration. Although it is an accepted view that urea has an effect on the properties of salt solutions, there is still a debate in the literature on how the urea molecule affects the properties of water. There are earlier experimental studies^5^ which claimed that urea–water solutions have non-ideal behavior, meaning that the water activity decreases with increasing concentration of urea. This view was contradicted by recent experimental studies^10^ and modelling^6^ where it is claimed that urea-water mixtures behave as ideal solutions.

Although the adsorption of urea on different surfaces, including functionalized silica and activated carbon, has been reported,^11,12^ to the best of our knowledge, it has not yet been investigated in urine. Human urine contains several odorant molecules which cause the typical odor of urine. Olfactory-GC-MS analysis on used incontinence products by Hall *et al.*^1^ revealed nine key odorants. For this study, p-cresol (4-methylphenol) was chosen as the odorant of interest. This odorant is one of the end products of the anaerobic microbial degradation of the amino acid tyrosine in the gut of humans, and the average level for urine of adults is 138.8 μM (range: 7.1–802 μM, corresponding to 0.76–87 ppm). The p*K*_a_ value of p-cresol is 10.3, rendering it uncharged at neutral pH and negatively charged at highly alkaline pH.

To reduce odor from urine, an efficient adsorbent is highly desired. Activated carbon (AC), is a popular material for adsorption in gas and solutions. Its popularity stems from its high surface area and relatively simple synthesize. The chemical and physical properties of the AC depend on its activation process. It generally consists of two differently hybridized carbon atoms: sp^2^ and sp^3^, the former as graphene sheets in the bulk material while the latter is mainly at the surface. Typical heteroatoms at the AC surface include nitrogen, oxygen, and hydrogen. Chemical treatment of AC can modify its surface properties, possibly enhancing its chemical reactivity towards adsorption. For example, NaOH exposure has been shown to increase the BET surface area and pore volume. HNO_3_ increases the number of acidic surface groups, lowering its point of zero charge (pH_PZC_) – the pH where the surface is neutral and having an equal number of positive and negative groups.^13^

To the best of our knowledge, no study has been reported in literature where the interactions of ions, urea, and odorant molecules with the activated carbon have been investigated experimentally or theoretically. Therefore, we have investigated the interactions of ions, urea, and the odorant molecule p-cresol with graphene surfaces in SU by utilizing molecular dynamics simulations. We have examined in detail the interactions with neutral (pristine and functionalized with carboxylic and hydroxyl groups) graphene and charged (60% deprotonated carboxylic and 50% deprotonated hydroxyl) referred as 50% charged surface and 100% deprotonated charged graphene oxide surfaces.

## 2. Methodology

### 2.1 Simulation methods

**Fig. 1:**
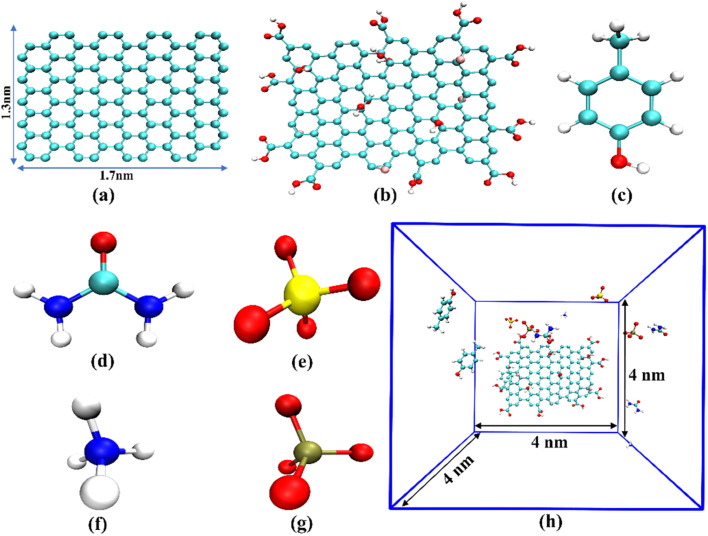
Snapshots representing the major constituents of simulation box (a) graphene; (b) graphene oxide; (c) p-cresol; (d) urea; (e) sulphate; (f) ammonium; (g) phosphate; (h) representative unit cell of the simulation, all the water molecules were removed for visual clarity.

Molecular dynamics (MD) simulations were used to explore the interactions among synthetic urine, odorant molecules (p-cresol), graphene, and its derivatives at an atomistic level. The size of the graphene (GRA) sheet considered in this study was 1.7 × 1.3 nm^2^ in size. The graphene oxide (GRO) sheet was modeled so that it consists of 10 carboxylate (COOH) at its edges and 6 epoxy (CO), 8 hydroxyl (OH) groups distributed randomly over its basal plane. The simulation box having the dimensions of 4 × 4 × 4 nm^3^ had been set up such that the sheets were fixed at the center of the box, with 3-molecules of p-cresol, 3-molecules of urea, 2-molecules of ammonium, phosphate, and sulphate ions each distributed randomly over the box. Requisite quantity of Na^+^(7), K^+^(4), Ca^2+^(2), Mg^2+^(3) cations and an equivalent number of Cl^−^(13) anions were added to the simulation box that was then filled with water molecules using the TIP4P model. Periodic boundary conditions were implemented in all three directions of the box to replicate an infinite system. In order to hold the GRA/GRO sheets at the center of the box, the carbon atoms of its surface were position-restrained, allowing the functional groups (COOH, CO, OH) to respond as per the dynamics of the system. The major constituents of a simulation box representing the interactions among graphene and its derivatives with synthetic urine are as shown in Fig. 1.

The simulations were performed using the GROMACS 2021.5 (ref. 14) package utilizing CHARMM forcefield. CHARMM forcefield parameters for graphene oxide, p-cresol, urea, sulphate, phosphate and ammonium ions are given in Table S1. All the initial structural co-ordinates and their topologies required for the simulation were generated using CHARMM-GUI.^15,16^ The initial structures were energy minimized appropriately to remove the bad contacts. To start with, the systems were equilibrated in two stages, first with NVT ensemble and then with NPT ensemble at a temperature of 300 K and a pressure of 1 atm for 100 ps each. After attaining the equilibration in temperature and pressure, the final production runs were performed using NVT ensemble for 50 ns. Thermodynamic equilibration of simulated systems: pristine graphene, graphene oxide with 0% deprotonation, graphene oxide with 50% deprotonation and graphene oxide with 100% deprotonation was achieved within the simulation time as shown in Fig. S1.

The temperature and pressure of the simulation box were controlled using a Berendsen^17^ thermostat with a relaxation time of *τ* = 0.1 ps and a barostat with *τ* = 2.0 ps. A time step of 2 fs was used for integrating the equations of motion. The radial and linear density distribution profiles calculated in this work are obtained using a bin width of 0.05 nm.

## 3. Results and discussions

### 3.1 Hydration of p-cresol and urea molecules in synthetic urine

**Fig. 2:**
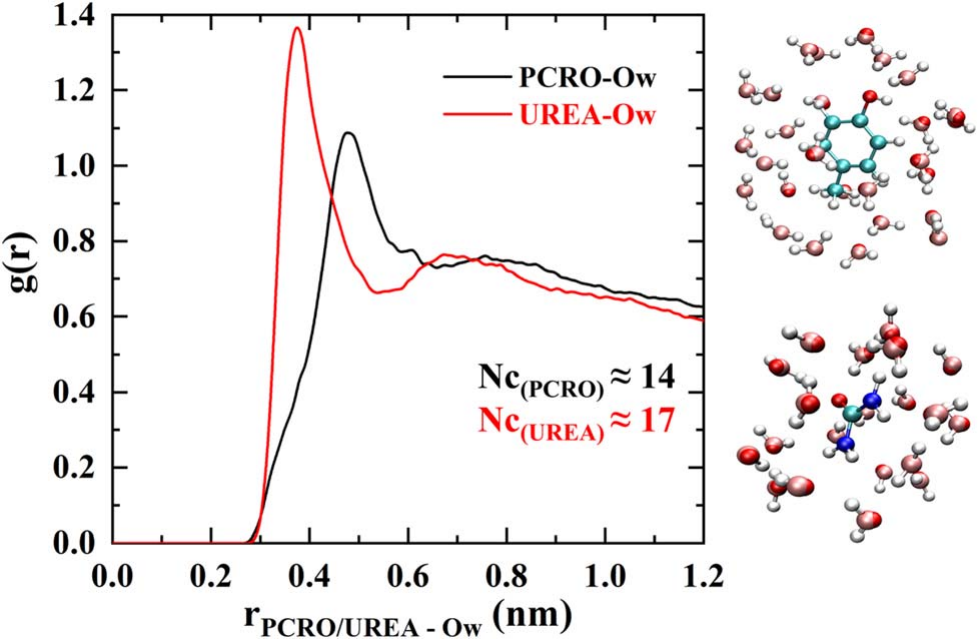
Radial distribution profiles and snapshots elucidating the hydration of p-cresol and urea molecules.

Synthetic urine is a complex mixture of ions and organic molecules, as shown in Table 1. To understand the interactions of ions and organic molecules with neutral and charged graphene surfaces, pure synthetic urine (SU) is considered as reference system with the odorant molecule p-cresol. To start with, MD simulations were performed for p-cresol in synthetic urine in a simulation box. Fig. 2 represents the distribution plot showing the hydration of p-cresol and urea molecules, normalized over the simulation duration of 50 ns. Two snapshots show the coordinating water molecules around the p-cresol and urea molecules.

From the RDF plot it can be seen that urea molecule is slightly more hydrated as compared to the p-cresol molecule. The urea is coordinated to 17 water molecules, as compared to the 14 for p-cresol. Moreover, from the RDF it is evident that the water molecules are coordinated closely to urea surface and have a pronounced distinct secondary peak compared to p-cresol. This might be because the urea molecule is smaller than p-cresol and cannot accommodate as many water molecules in its primary hydration shell. Therefore, a second hydration shell is created. In literature, experimentally determined hydration numbers of urea vary depending on the method of determination. The hydration number determined by compressibility measurements was 13,^18^ and when determined by NMR,^19^ IR,^20^ and neutron scattering,^21^ it was 7. Usually, MD simulations and other methods provide information about how many water molecules surround the molecule, represented as its coordination number (CN or N_c_). However, it would be interesting to know how tightly the water molecules are bound to urea and p-cresol. Such information can be obtained by dielectric spectroscopy. Recently, Agieienko and Buchner^22^ have performed such measurements on urea solutions. They found that there were only two water molecules strongly bonded with each urea molecule, and this number decreased as the urea concentration increased. Moreover, they found that urea has a significant effect on the reorientation dynamics of the water molecules, which is also in agreement with the MD simulations.^8^ The hydration of p-cresol has also been investigated to some extent by experimental and theoretical methods. In the *ab initio* study, it was found that water act as H-bond donor to the hydroxyl group of p-cresol,^23^ whereas an NMR and Rayleigh scattering study^24^ indicated that there are 4 water molecules coordinated. Unfortunately, there is no dielectric spectroscopy data available for p-cresol from which one could find the number of water molecules that are tightly bounded to each p-cresol molecule.

### 3.2 Ion interactions with neutral and charged graphene interfaces

**Fig. 3:**
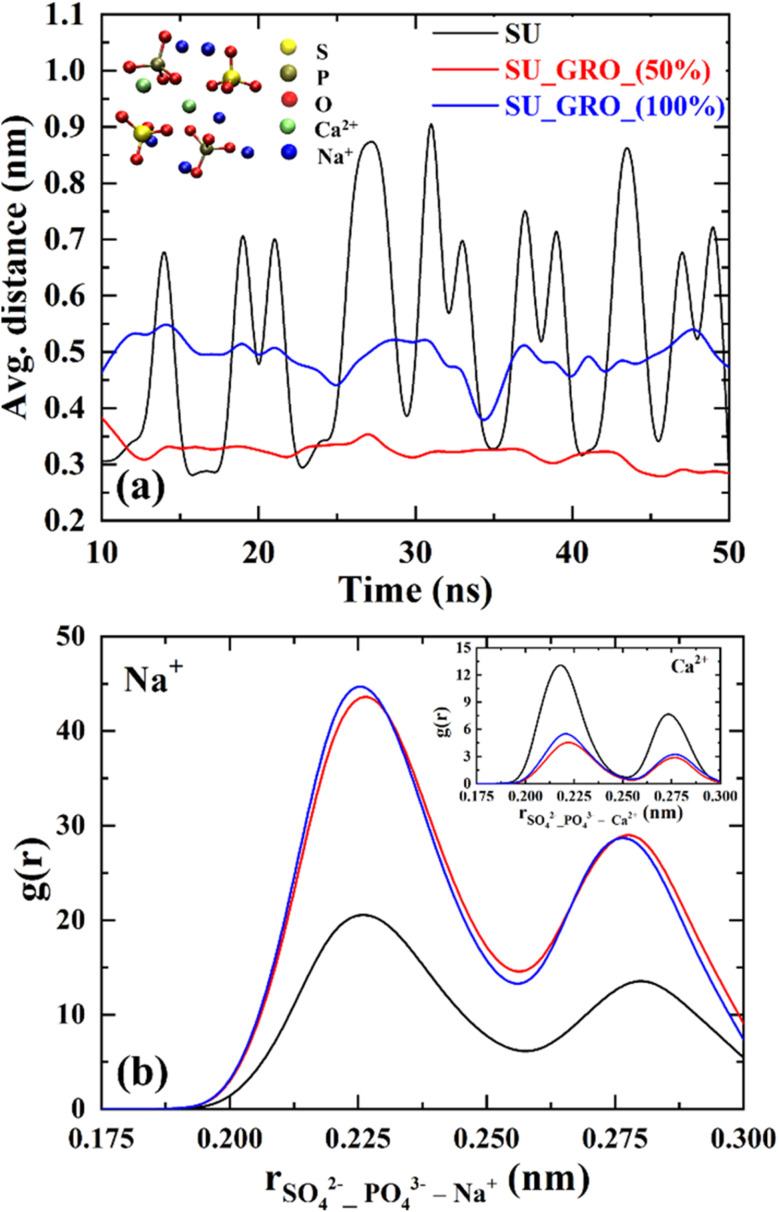
(a) Ion complexation among PO_4_^3−^ and SO_4_^2−^ mediated by Ca^2+^ and Na^+^ counterions of synthetic urine (SU); (b) radial number density distribution of Na^+^ and Ca^2+^ (inset figure) ions in between the PO_4_^3−^ and SO_4_^2−^ complexes.

Presence of surface neutral or charged can have significant effect on the distribution of ions in complex solution such as urine. To explore this we have investigated the cluster formation of ions present in synthetic urine, *i.e.*, PO_4_^3−^ and SO_4_^2−^, Ca^2+^ and Na^+^ in the presence of neutral but functionalized graphene oxide as well as negatively charged graphene oxide surfaces. In Fig. 3a the distances between the phosphate and sulphate ions from their respective center of masses averaged over the simulation duration of 50 ns are shown, whereas in Fig. 3b the radial density distribution of Na^+^ and Ca^2+^ ions within complexes are shown.

Interestingly, stable complexation between SO_4_^2−^, PO_4_^3−^ mediated by Ca^2+^ and Na^+^ ions are seen specifically in the systems with charged graphene surfaces compared to the pure synthetic urine. We note, however, that there are fewer Na^+^ ions in the pure synthetic urine compared to synthetic urine with charged surfaces because Na^+^ ions were added to neutralize the charged groups on surface. As shown in Fig. 3a, the average distance between the center of mass of sulphate and phosphate ions is ≈0.35 nm in case of 50% charged GRO surface and ≈0.45 nm with 100% charged GRO surface leading to less fluctuations compared to pure synthetic urine. The electrostatic forces are responsible for bridging the two similarly charged anions by the cations, therefore, as the number of Na^+^ ions increased in the case of charged surfaces the stability of cation mediated complexation increases. Fig. 3b shows the number density distribution of Na^+^ and Ca^2+^ ions associated among SO_4_^2−^ and PO_4_^3−^ complexes in radial 3 Å distance. Larger accumulations of Na^+^ and Ca^2+^ near the anions is clearly seen leading to stabilization of complexes. Note that water is omitted around the ions for the sake of clarity. Such a strong interaction between lipid phosphate groups with Ca^2+^ and Na^+^ has also been observed experimentally in a recent study by van der Post *et al.*.^25^ It is also well-known from the salt solutions that Na^+^ and SO_4_^2−^ have a strong affinity for forming ion pairs.^26^ If such strong complexation between multivalent anions and cations prevails even in the presence of a solid surface, it can have a significant effect on the adsorption of odorant molecules. For example, strong complexation will retain the cations in a complexed form, meaning that few counter-ions will be available for the charged surface, which can enhance the adsorption of p-cresol.

Activated carbon (AC) is an efficient adsorbent for a wide range of molecules as well as for ions. Creating an atomistic-level model for activated carbon is a formidable task due to its complex porous structure. Therefore, in this study, we have chosen to depict it as pristine graphene (GRA) and graphene oxide (GRO) sheets with carboxylic, epoxy and hydroxyl functional groups. This study aims to investigate the interaction of p-cresol and urea molecules with graphene and its derivatives in synthetic urine (SU) using MD simulations. Even though this graphene-based system is the simplest one can think of, it is computationally less demanding for atomistic MD simulations.

**Fig. 4:**
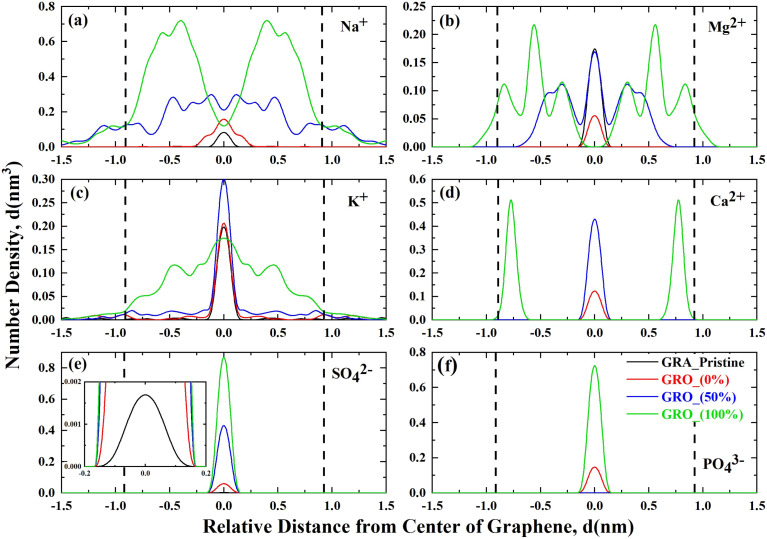
Linear density distribution plots representing the ion condensation at the interface of neutral and charged graphene surfaces: (a) sodium; (b) magnesium; (c) potassium; (d) calcium; (e) sulphate and (f) phosphate ions; the dotted lines represent the edges of the graphene/graphene oxide sheets in the *X*-direction of simulation box. GRA_Pristine (unfunctionalized graphene), GRO (functionalized graphene) and % (percentage of charge on surface).

In Fig. 4 the distributions of ions near neutral sheets (graphene and graphene oxide with protonated carboxylic and hydroxyl groups) and charged sheets (graphene oxide with 50 and 100% de-protonation) are shown.

**Fig. 5:**
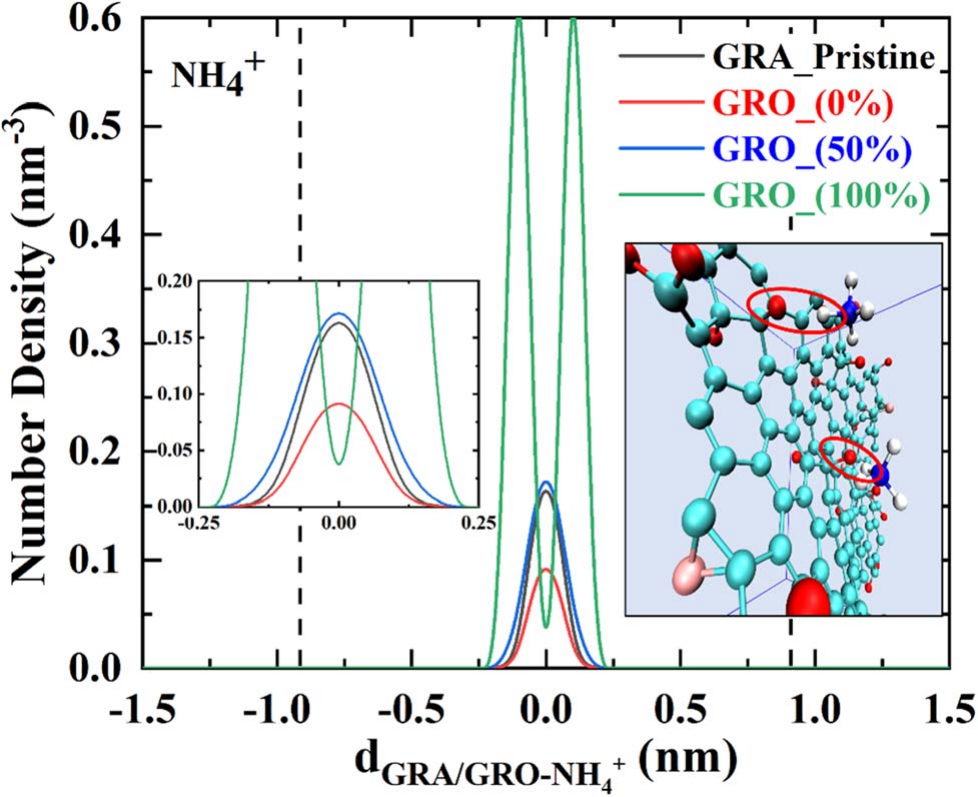
Snapshot of MD simulation showing the interaction of NH_4_^+^ with the deprotonated hydroxyl groups of GRO (100%). Linear density distribution of the NH_4_^+^ ion representing its interaction with the interface of GRA/GRO at different conditions considered in this study. GRA (unfunctionalized graphene), GRO (functionalized graphene) and % (percentage of charge on surface).

Sodium ions near the neutral surfaces, *i.e.*, the pristine graphene and functionalized graphene surfaces, show no preferential interaction with the protonated functional groups. In a recent study^27^ sum frequency generation (SFG) spectroscopy along with MD simulations were used to study the interaction of neutral graphene oxide surface having carboxylic groups with the alkali monovalent ions. It was found that only Li^+^ due to its strong hydration shell showed different interaction behavior than other monovalent alkali ions including Na^+^. These findings are in accordance with what we have observed in our simulation of uncharged graphene surface interactions with monovalent Na^+^ ions. However, the charging of the surface has a significant effect, as ions accumulate near the edges of the graphene sheet where the deprotonated functional groups reside (see Fig. 1 in the MD section). As expected, the 100% charged GRO surface accumulates more Na^+^ ions than the 50% charged. The potassium and ammonium ions show similar interactions with the neutral graphene as seen for the sodium ions; they accumulate near the middle of the sheet. However, there are significant differences in the interaction with the charged surfaces. Potassium ions seem to accumulate near the center of the surface when half of the surface groups are deprotonated, whereas, in the case of fully charged surface, there is a broader distribution with distinct accumulation at the edges. Such accumulation at edges is even more distinct for NH_4_^+^ ions at the 100% charged surface (Fig. 5). Furthermore, from the linear density plot, it can be deduced that NH_4_^+^ ions specifically interact with the deprotonated hydroxyl groups to a larger extent than the carboxylic groups because they reside within the −0.75 to +0.75 nm region, where the hydroxyl groups are. This is further highlighted by a snapshot in Fig. 5.

Sodium ions do not show such strong preferred interaction and are complexed with both deprotonated hydroxyl and carboxylic groups. Potassium ions, on the other hand, show a mixed behavior, which is a specific interaction with the hydroxyl groups at 50%, and complexation with both carboxyl and hydroxyl at 100% deprotonated surface. In a previous study by one of the authors,^28^ as well as in dielectric spectroscopy studies,^29^ it has been shown that, in water, the K^+^ and NH_4_^+^ ions in their chloride salts are very weakly hydrated, and the water around them is highly mobile. This weak hydration, in turn, determines the interaction with charged groups. Recently, Vlachy *et al.*^30^ simulated the interaction of carboxylic groups with K^+^ and NH_4_^+^ and found that K^+^ interacts more strongly than NH_4_^+^. Van der Vegt^31^ has also pointed that the interaction order of alkali cations with -COO^−^ group is NH_4_^+^ < K^+^ < Li^+^ < Na^+^. Moreover, it was also mentioned that Na^+^ interacts strongly with PO_4_^3−^, forming solvent-shared ion pairs, while no such pairs were observed for K^+^ and NH_4_^+^. These results pin point that the ion hydration plays a significant role in forming stable ion pairs as well in determining the interaction with charged groups on surfaces.

The interaction of Mg^2+^ and Ca^2+^ with neutral graphene surfaces follows the same behavior as monovalent ions, *i.e.*, an accumulation near the middle of the sheet in the case of a neutral surface (Fig. 4). However, distinct features appear in the interaction with the charged graphene surfaces. Both Ca^2+^ and Mg^2+^ ions specifically interact with the charged groups and accumulate near the edges where the charged groups reside. However, a distinctive feature in the Mg^2+^ interaction, compared to Ca^2+^, is that it has clear, distinct peaks showing the interaction with both carboxylic as well as with hydroxyl groups. In comparison, Ca^2+^ ions are preferentially complexed with the hydroxyl groups. Moreover, distribution peaks of Mg^2+^ are broader than Ca^2+^ indicating that Mg^2+^ is more hydrated than Ca^2+^ even in the complexed form.

An error analysis was performed by calculating the standard deviation of ion distances accumulated over 2500 simulation frames to quantify the temporal variability of ion positions with respect to the graphene surfaces as shown in Fig. S2.

In general, the fluctuations are larger in regions closer to the graphene surface, particularly near the first density peak. This indicates that ions within the interfacial layer experience stronger local rearrangements and transient adsorption–desorption events. These fluctuations are a consequence of competing interactions between the ions, solvent molecules, and surface functional groups of the graphene sheets. In contrast, the regions away from the interface (bulk region) show much smaller error values, implying that ions there are more uniformly distributed and undergo primarily diffusive motion with minimal structural confinement.

A comparison between the pristine and deprotonated graphene sheets further shows that the deprotonated surface exhibits slightly reduced ion fluctuations, suggesting more stable ion interaction due to the presence of negatively charged functional groups. This stabilization effect aligns with the observed enhancement in local ion density near the deprotonated surfaces.

**Fig. 6:**
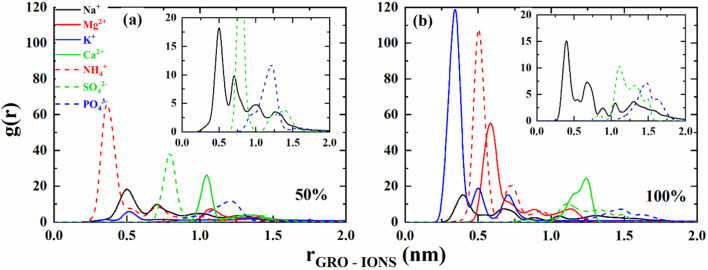
Radial distribution profiles representing the ion condensation at the interface of graphene oxide; (a) 50% charged graphene oxide; (b) 100% charged graphene oxide (GRO: functionalized graphene).

To highlight further the ion condensation near the charged graphene sheets, radial distribution functions were calculated starting from the center of the graphene sheet moving radially outward with a bin width of 0.05 nm. The RDFs were normalized by the total concentrations of the respective ions in the simulation box. The normalized RDFs for the ion condensation near the 50% and 100% charged surfaces are shown in Fig. 6a and b, respectively.

It is evident from the above plots that the extent of charging has a significant effect on ion condensation near the GRO surface. In the case of the 50% charged GRO surface, the NH_4_^+^ ions approach closest to its interface, while the Na^+^ and K^+^ ions approach at the same distance from the surface. It is well known from the theoretical and experimental data^28,29^ that Na^+^ ions are more hydrated than K^+^ and NH_4_^+^. Moreover, experimental measurements, such as zeta potentials, potentiometric titrations, and aggregation of colloids, also indicate that weakly hydrated ions approach closer to the surface than strongly hydrated ions, resulting in the reduction in the zeta potential and faster aggregation.^32–34^ In our recent study we have shown that Na^+^ ions lose their hydrating molecules approaching negatively charged silica surface whereas Mg^2+^ keeps its hydration shell intact.^33^ Interaction of Ca^2+^ and Mg^2+^ with the 50% charged surface follows the hydration argument, such that, due to the weak hydration of Ca^2+^, it can approach closer to the surface than the strongly hydrated Mg^2+^.

The ion condensation near the 100% charged GRO surface shows distinct features as compared to the 50% charged GRO surface. The monovalent K^+^ ions approach closest to the surface, followed by NH_4_^+^ and Na^+^ ions. This result can be rationalized that when surface is highly charged, it attracts strongly the weakly hydrated counterions, such as K^+^ and NH_4_^+^. However, the ion condensation of Mg^2+^ and Ca^2+^ near the 100% charged GRO surface showed the opposite behavior from the 50% charged surface. Mg^2+^ approaches much closer to the surface than Ca^2+^ ions with the fully charged graphene surface. Such a reversal in the interaction of divalent cations with the negatively charged surface has recently been reported for the gelling of silica in MgCl_2_ and CaCl_2_ at different pH values. At pH 7, the Ca^2+^ ions adsorb strongly on the weakly charged silica surface, but at pH 9, when the silica surface is highly charged, Mg^2+^ adsorbs strongly, giving rise to a shorter gel-time.^33^ In another study on silica surface interaction with divalent ions,^35^ it was shown by simulations that strong ion pairs are formed with Mg^2+^ at the negatively charged silica–solution interface, compared to Ca^2+^ and Ba^2+^.

In a recent study^36^ by one of the authors are it was shown that polarization of water molecules coordinating small highly charged ions such as Mg^2+^ is the reason that there are solvent separated ion pairs in MgSO_4_ salt solutions. On the other hand, due to relatively weak polarization of water coordinating Ca^2+^ ions there are contact ion pairs in CaSO_4_ salt solutions. Stoher *et al.*^37^ have also shown, by performing sum frequency generation spectroscopy and molecular simulations, that when a monolayer of carboxylic groups is weakly charged, K^+^ is more strongly complexed than Li^+^. However, when the monolayer is highly charged, Li^+^ interacts stronger than K^+^. The rationale for this reversed affinity was given due to the homogeneous, strong hydration layer at the fully charged surface compared to the discrete hydration layer at a weakly charged surface. The homogeneous hydration layer enhances the affinity of strongly hydrated counterions, such as Li^+^, by forming solvent-separated ion pairs. We can invoke the same argument here that when the graphene oxide is 100% charged, it has a strong affinity for the strongly hydrated Mg^2+^ because surface and counterions have matching water structures which along with electrostatic interactions create such strong interaction.

### 3.3 Hydration of graphene/graphene oxide interface

**Fig. 7:**
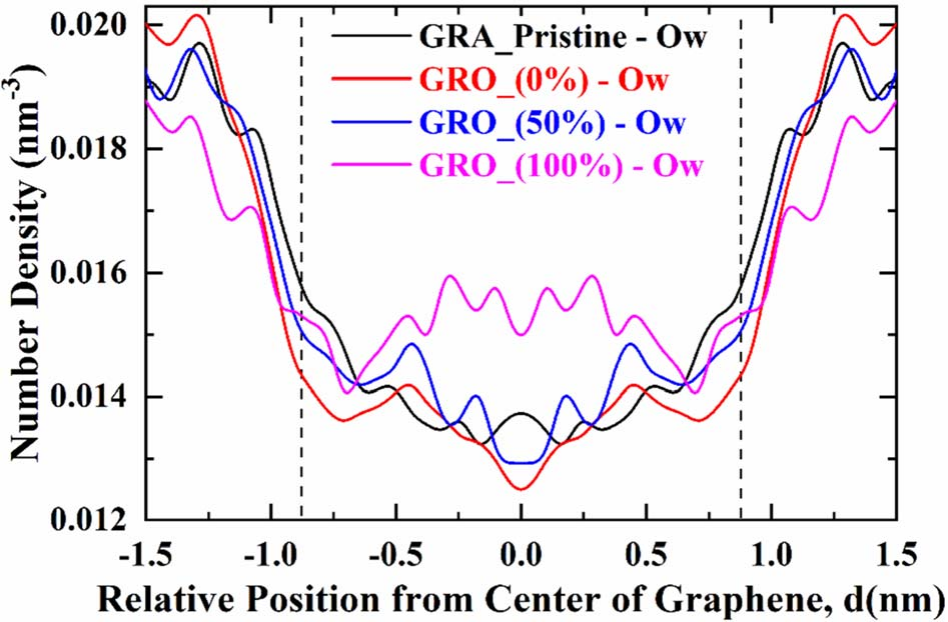
Number density of water molecules depicting the hydration of GRA/GRO surfaces. GRA (unfunctionalized graphene), GRO (functionalized graphene) and % (percentage of charge on surface).

The extent of the hydration of the GRA/GRO surface can also affect the adsorption mechanism of p-cresol. Fig. 7 shows the number density distribution of water molecules accumulated at the interface of the neutral and charged graphene surfaces leading to the formation of a hydration layer. The number density distribution is calculated such that the simulation box is sliced linearly in the direction of the *X* axis using a bin width of 0.05 nm with limitations in the *X*, *Y*, and *Z* directions.

It can clearly be seen that the number density of water molecules at the 100% charged GRO surface is much higher than at the 50% charged GRO surface as well as the neutral GRA/GRO surfaces. The highest water density difference is found in the basal plane of the surface. This result, at first sight, sounds counter-intuitive, but we should keep in mind that charged groups are resided at the edges and will accumulate water due to hydration. Hydrogen bonding with other water molecules brings many more water molecules near the surface. This will result in an increased number density of water molecules near the whole surface.

The hydration layer observed at the interface of the 50% charged GRO surface is inhomogeneous due to the unequal distribution of charged groups (deprotonated hydroxyl groups) on its basal plane. Since only half of the groups are hydrated, there will be a discrete hydration layer near the surface and will resemble the uncharged surface. There are, of course, more hydrating molecules near the 50% charged than uncharged surface, as can be seen from the plot but not as many as in the case of a 100% charged surface.

### 3.4 Interactions of p-cresol and urea with neutral and charged graphene interfaces

**Fig. 8:**
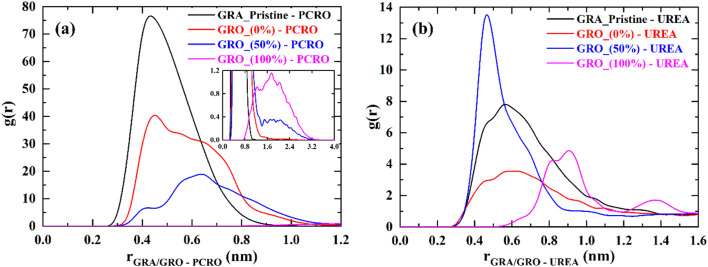
(a) p-cresol and (b) urea interactions with GRA/GRO sheets in synthetic. GRA (unfunctionalized graphene), GRO (functionalized graphene), % (percentage of charge on surface) and PCRO (p-cresol).

The interactions of p-cresol and urea with neutral and charged graphene surfaces are shown in the form of radial densities in Fig. 8a and b, respectively.

**Fig. 9:**
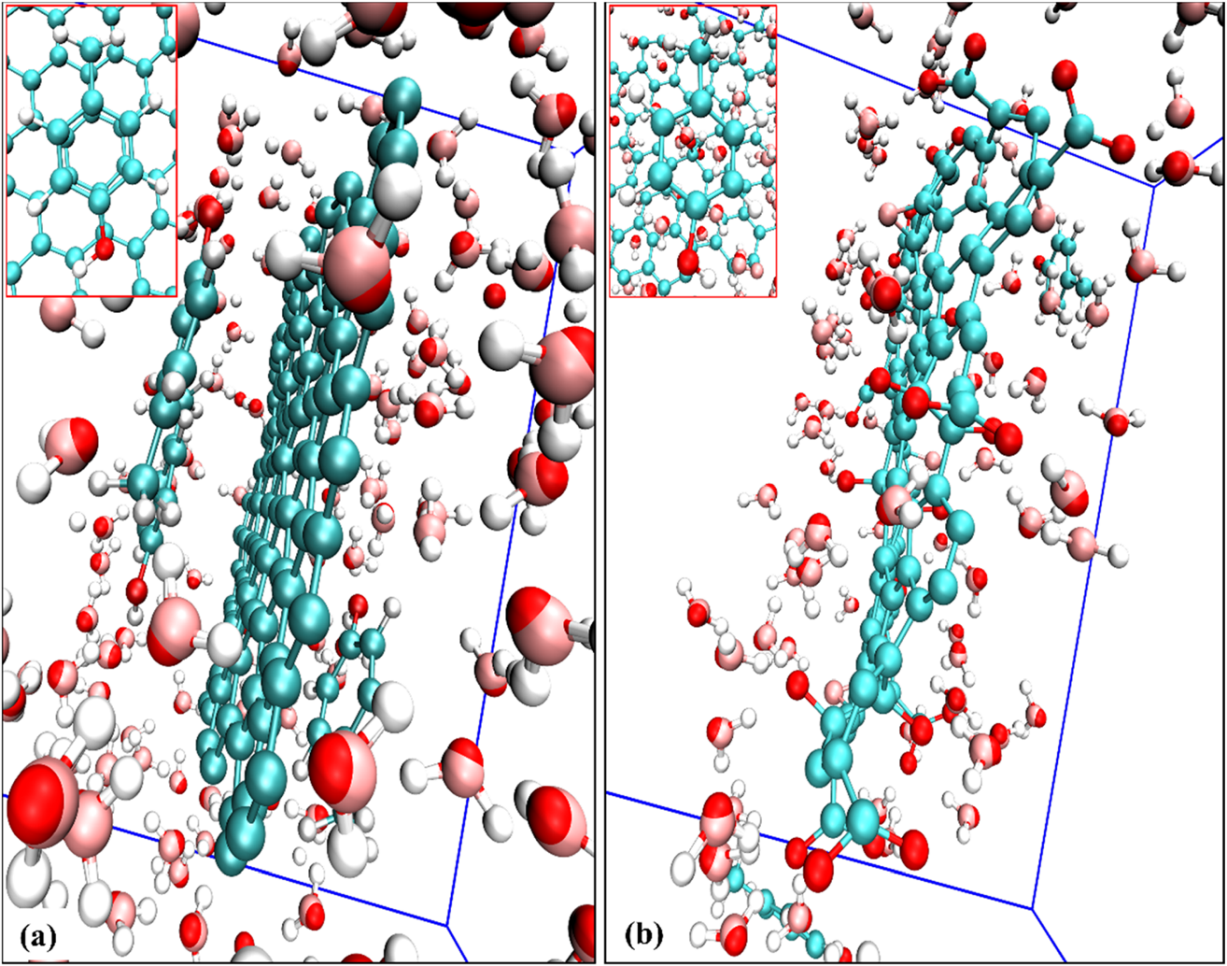
Snapshots illustrating the water-mediated interaction of p-cresol molecules in the vicinity of (a) pristine graphene sheet and (b) 100% deprotonated graphene oxide sheet. The insets highlight the water molecules located between the neutral/charged graphene sheets and the p-cresol molecule.

Since p-cresol is modelled as protonated and neutral, the strongest interaction is found with the pristine graphene surface. The interaction of p-cresol becomes weaker as the surface acquires functional groups and a negative charge. On the 100% charged GRO surface, no adsorption is seen. The driving forces for the enhanced interaction between p-cresol and pristine graphene are the van der Waals attractive forces. Since the pristine graphene surface is weakly hydrated, the hydration has a negligible effect on the interaction of p-cresol (Fig. 9a). However, as the surface obtains functional groups, its hydration increases (Fig. 9b), and the accessible graphene surface area decreases and, consequently, interactions with p-cresol are weakened. On the charged surface, its hydration along with the accumulation of counter-ions near the surface make it difficult for neutral p-cresol molecules to interact. Therefore, on the 50% charged surface, the p-cresol molecules are repelled from the surface compared to the functionalized uncharged surface. When the surface is 100% charged, the hydration layer on the GRO surface and the water brought to the interface by counter-ions is so thick that there is negligible p-cresol accumulation at the interface.

Compared to p-cresol, the interaction of urea with neutral and charged GRA/GRO surfaces does not show a successive decrease in the extent of interaction as the surface becomes charged. The interaction of urea with GRA is much weaker than p-cresol due to its smaller size, resulting in weak van der Waals interactions with the surface. However, interaction of urea with charged surface shows mixed behaviour *i.e.*, stronger with the 50% charged graphene surface compared 100% charged surface. It is evident from the RDF plot for the 100% charged GRO surface that urea molecules are pushed away from the surface, and this can be due to the significant hydration layer on its surface, in addition to the water molecules brought to the interface by counter-ions.

The interaction behavior of urea observed for the 50% charged GRO surface is difficult to explain. Note that we have repeated those simulations but got the same result. One possibility is that since urea is a small molecule and when the GRO surface is weakly charged, there are discrete charged groups on the surface. This means the water structure on the GRO surface can be discrete and not a continuous hydrated layer, as shown in Fig. 7. This, in turn, can lead to the urea molecule directly interacting with the charged surface groups, bringing molecules closer to the surface. Fundamental mechanisms driving the urea adsorption on charged solid surfaces have yet to be resolved because in some studied it is claimed that deprotonated carboxylic groups present on the activated carbon play significant role^38^ whereas in another study^39^ it is claimed that the negatively charged oxygen of deprotonated hydroxyl group form the bond with amide group of urea. In an other recent study^40^ iron based metal organics-frameworks MIL-101 was used to adsorb the urea from solution and a superfast adsorption was observed. It was pointed out that the very high surface area of MIL-101 played significant role in the adsorption of urea compared to the negative charge present on the surface.

**Fig. 10:**
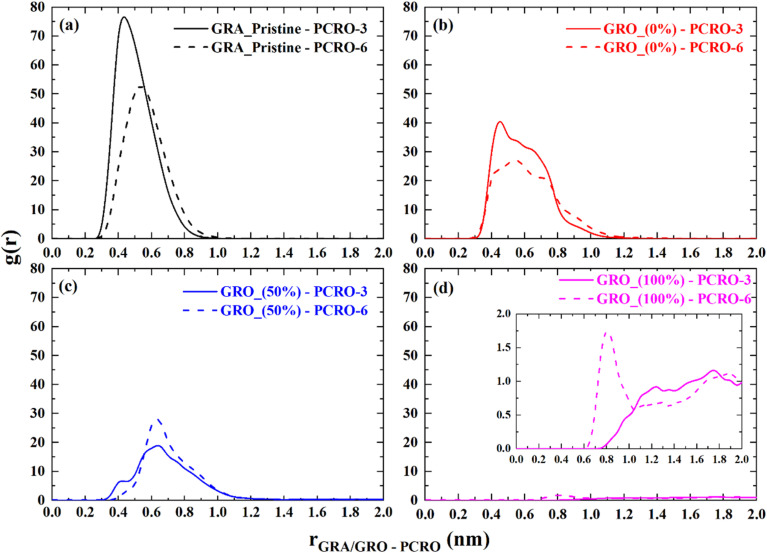
Radial distribution profiles representing the interaction of odorant molecule, p-cresol with GRA/GRO interfaces at different p-cresol concentrations. (a) Pristine graphene; (b) graphene oxide with 0% deprotonation; (c) graphene oxide with 50% deprotonation; (d) graphene oxide with 100% deprotonation. The abbreviation PCRO-3 and PCR-6 stands for 3 and 6 number of p-cresol molecules respectively. GRA (unfunctionalized graphene), GRO (functionalized graphene) and % (percentage of charge on surface).

To explore how the concentration of p-cresol will affect the interaction with the GRA/GRO interfaces, the number of p-cresol molecules were doubled from 3 to 6 in the simulation box, and the results are shown in Fig. 10. These two cases correspond to p-cresol concentrations of 50 and 100 ppm, respectively.

Interestingly, with the neutral GRA/GRO surfaces, there is a slight decrease in the interaction of p-cresol molecules as the number of molecules are increased from 3 to 6 in the simulation box. This can be rationalized because when the number of molecules is increased, there will be enhanced van der Waals interactions between the p-cresol molecules. On the charged surface, the interaction between p-cresol molecules and surface becomes weaker. It is evident from Fig. 10 that the intensity of g(r) peak reduces considerably near the 50 and 100% charged surface compared to pristine and neutral surfaces. Furthermore p-cresol molecules are pushed away from the surface. This is result of accumulation of counter-ions near the charged surfaces. To highlight the mechanism underlying the concentration dependent p-cresol interactions with neutral and charged surfaces RDF plots among p-cresol molecules were calculated as shown in Fig S3. In the case of 6 p-cresol aggregates are formed with persistent structure in the presence of neutral as well as charged surfaces. However, in the case of 3 p-cresol the aggregates do not have persistent structures. This means that the total van der Waals interaction between aggregates and surface will be enhanced in the case of 6 p-cresol than 3 p-cresol. This is clearly evident in the case of 50 and 100% charged surface where 6 p-cresol show sharp peaks whereas 3 p-cresol show wide distribution. Naturally question arises if there are strong van der Waals interaction between surface and 6 p-cresol why don't we see enhanced interaction with the pristine and neutral graphene surfaces. What is evident from Fig. 10 is that the intensity of interaction with functionalized but neutral surface is decreased compared to the pristine surface. This is due to the relatively stronger hydration of functionalized surface than neutral surface. The enhanced interaction intensity in the case 3 p-cresol with the pristine surface compared to 6 p-cresol is due to fact that 3 p-cresol molecules do not form aggregates and freely can interact with the surface. Since, hydration of surface is negligible p-cresol molecules can have direct interaction with the surface which is exhibited as enhanced intensity.

**Fig. 11:**
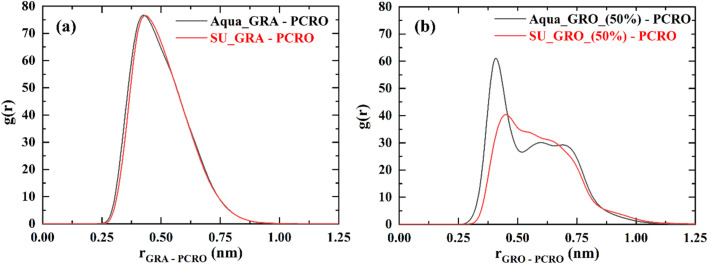
Radial distribution functions of p-cresol in water and synthetic urine with (a) pristine GRA sheet and (b) 50% charged GRO sheet. GRA (unfunctionalized graphene), GRO (functionalized graphene), % (percentage of charge on surface) and PCRO (p-cresol).

To further investigate the influence of ions on the interaction of p-cresol with the neutral GRA and 50% charged GRO surfaces is affected by the synthetic urine, simulations in pure water and synthetic urine were performed. Note that in these simulations 3 p-cresol molecules were used because in all simulations except where concentration effects were explored only 3 p-cresol molecules were used (Fig. 11).

In the case of pristine graphene, the interaction of p-cresol in water and synthetic urine is similar. This is understandable because ions present in synthetic urine do not accumulate near the pristine graphene and therefore have no effect on the p-cresol interaction. On the other hand, the interaction with the 50% charged GRO surface in synthetic urine is much weaker showing a broad distribution. The weakening of the p-cresol interactions is due to the accumulation of ions as well as the hydration layer formed near the charged surface (Fig. 6 and 7), pushing away the p-cresol molecules from the surface.

## 4. Conclusions

The presented molecular dynamics simulations of graphene based nanostructures in synthetic urine with the odorant molecule p-cresol have shown that the ionic composition of the solution and the sheet's surface charge have profound effect on the p-cresol interactions. The MD simulations revealed that PO_4_^3−^, SO_4_^2−^, Ca^2+^, and Na^+^ form clusters with persistent structures during the whole simulation even in the presence of negatively charged surface. It was found that urea is slightly more hydrated than p-cresol in synthetic urine which is in accordance with the measured solubilities of urea and p-cresol in water. The MD simulations have also revealed that ions have a specific affinity for the surface groups. NH_4_^+^ ions were closely associated with the negatively charged oxygen of the deprotonated hydroxyl groups during the whole simulation, whereas Na^+^ ions were associated with the negatively charged oxygens of both the hydroxyl and carboxylic groups. Divalent Mg^2+^ ions were complexed with both the functional groups, while Ca^2+^ ions are selectively complexed with the hydroxyl groups. Radial density profiles of the ion distribution near the surface revealed that weakly hydrated ions are attracted to the surface as the surface gets negatively charged. However, this was not the case for the divalent Mg^2+^ and Ca^2+^ ions. At the 50% charged surface, Ca^2+^ was nearer to the surface than Mg^2+^ whereas in the case of 100% charged surface, it was the opposite. A plausible explanation of this trend reversal has been put forward *i.e.*, when surface is highly charged it has a homogeneous strong hydration layer which in turn has higher affinity for strongly hydrated ions through solvent-shared ion pair formation.

From the MD simulations results it became clear that p-cresol strongly interacts with the neutral graphene surfaces in synthetic urine compared to the charged surfaces. Moreover, the interaction of p-cresol becomes weaker as the surface charge increases. This is due to the accumulation of counter-ions near the charged surface. In contrary to p-cresol, the interaction of urea with the 50% charged surface is stronger than with the neutral surface. A plausible explanation of this odd result is that the discreteness of the hydration layer on the 50% charged surface gives rise to such directed affinity. Simulations also revealed that the p-cresol interaction with the 50% charged surface is stronger in pure water compared to synthetic urine.

## Conflicts of interest

Authors of this work have no conflict of interest.

## Supplementary Material

RA-015-D5RA06390F-s001

## Data Availability

The dataset supporting the findings of this study with key input files (.pdb/.gro, .itp, .top, .mdp) and output files (.tpr, .xtc) from the molecular dynamics simulations is available in the repository of Zenodo at DOI: https://doi.org/10.5281/zenodo.17498391. Supplementary information: the force field parameters of all the molecules and ions utilized in the simulations. Plots illustrating the thermodynamic equilibration of the simulated systems. Linear density distribution profiles representing ion condensation at the interfaces of neutral and charged graphene surfaces along with their standard deviations. Radial distribution plots depicting the aggregation behavior of p-cresol molecules at varying concentrations across the systems considered in this study. See DOI: https://doi.org/10.1039/d5ra06390f.
