# Supplementary material for: Interactions of ions and odorant molecules with graphene-based nanostructures in synthetic urine: a molecular dynamics exploration
Source: RSC Adv. 2025 Nov 7;15(51):43334–44. doi: 10.1039/d5ra06390f (PMC12593198; doi:10.1039/d5ra06390f)
Supplement: RA-015-D5RA06390F-s001 [file RA-015-D5RA06390F-s001.pdf]

Supplementary Information (SI) for RSC Advances.
This journal is © The Royal Society of Chemistry 2025

Supplementary Information

Interactions of ions and odorant molecules with graphene-based nanostructures in synthetic urine: A molecular dynamics exploration

Prasad Rama[a], Isabelle Simonsson[a], Zareen Abbas[a,*]

[a]Department of Chemistry and Molecular Biology

University of Gothenburg, Gothenburg – 41125, SWEDEN

***Email:** zareen.abbas.gu.se

Table S1: CHARMM forcefield parameters for Graphene oxide, p-cresol, Urea, Sulphate, Phosphate and Ammonium Ions

Ions/Molecules	Atom Name	Mass	Charge	Sigma	Epsilon
**Graphene**	CG2R61	12.0110	-0.120	3.55005321205e-01	2.928800e-01
**Graphene Oxide**	CG2DC1	12.0110	-0.152	3.72395664183e-01	2.845120e-01
	CG2DC2	12.0110	-0.161	3.72395664183e-01	2.845120e-01
	CG2O2	12.0110	0.668	3.02905564168e-01	4.100320e-01
	CG2R61	12.0110	0.137	3.55005321205e-01	2.928800e-01
	CG2R67	12.0110	-0.045	3.55005321205e-01	2.928800e-01
	CG301	12.0110	0.233	3.56359487256e-01	1.338880e-01
	CG3RC1	12.0110	0.106	3.56359487256e-01	1.338880e-01
	HGA1	1.0080	0.090	2.38760856462e-01	1.882800e-01
	HGA4	1.0080	0.124	2.22724679535e-01	1.297040e-01
	HGP1	1.0080	0.439	4.00013524445e-02	1.924640e-01
	HGR61	1.0080	0.115	2.42003727796e-01	1.255200e-01
	OG2D1	15.9994	-0.357	3.02905564168e-01	5.020800e-01
	OG311	15.9994	-0.671	3.14487247504e-01	8.037464e-01
	OG3C31	15.9994	-0.278	2.93996576986e-01	4.184000e-01
**Urea**	CG2O6	12.0110	0.600	3.56359487256e-01	2.928800e-01
	HGP1	1.0080	0.340	4.00013524445e-02	1.924640e-01
	NG2S2	14.0070	-0.690	3.29632525712e-01	8.368000e-01
	OG2D1	15.9994	-0.580	3.02905564168e-01	5.020800e-01

**p-cresol**	CG2R61	12.0110	0.110	3.55005321205e-01	2.928800e-01
	CG331	12.0110	-0.270	3.65268474438e-01	3.263520e-01
	HGA3	1.0080	0.090	2.38760856462e-01	1.004160e-01
	HGP1	1.0080	0.420	4.00013524445e-02	1.924640e-01
	HGR61	1.0080	0.115	2.42003727796e-01	1.255200e-01
	OG311	15.9994	-0.530	3.14487247504e-01	8.037464e-01
**SO_4**	OG2P1	15.9994	-0.567	3.02905564168e-01	5.020800e-01
	SG3O1	32.0600	0.268	3.74177461619e-01	1.966480e+00
**PO_4**	PG2	30.9738	0.680	3.83086448800e-01	2.447640e+00
	OG2P1	15.9994	-0.920	3.02905564168e-01	5.020800e-01
**NH_4**	NG3P3	14.0070	-0.320	3.29632525712e-01	8.368000e-01
	HGP2	1.0080	0.330	4.00013524445e-02	1.924640e-01

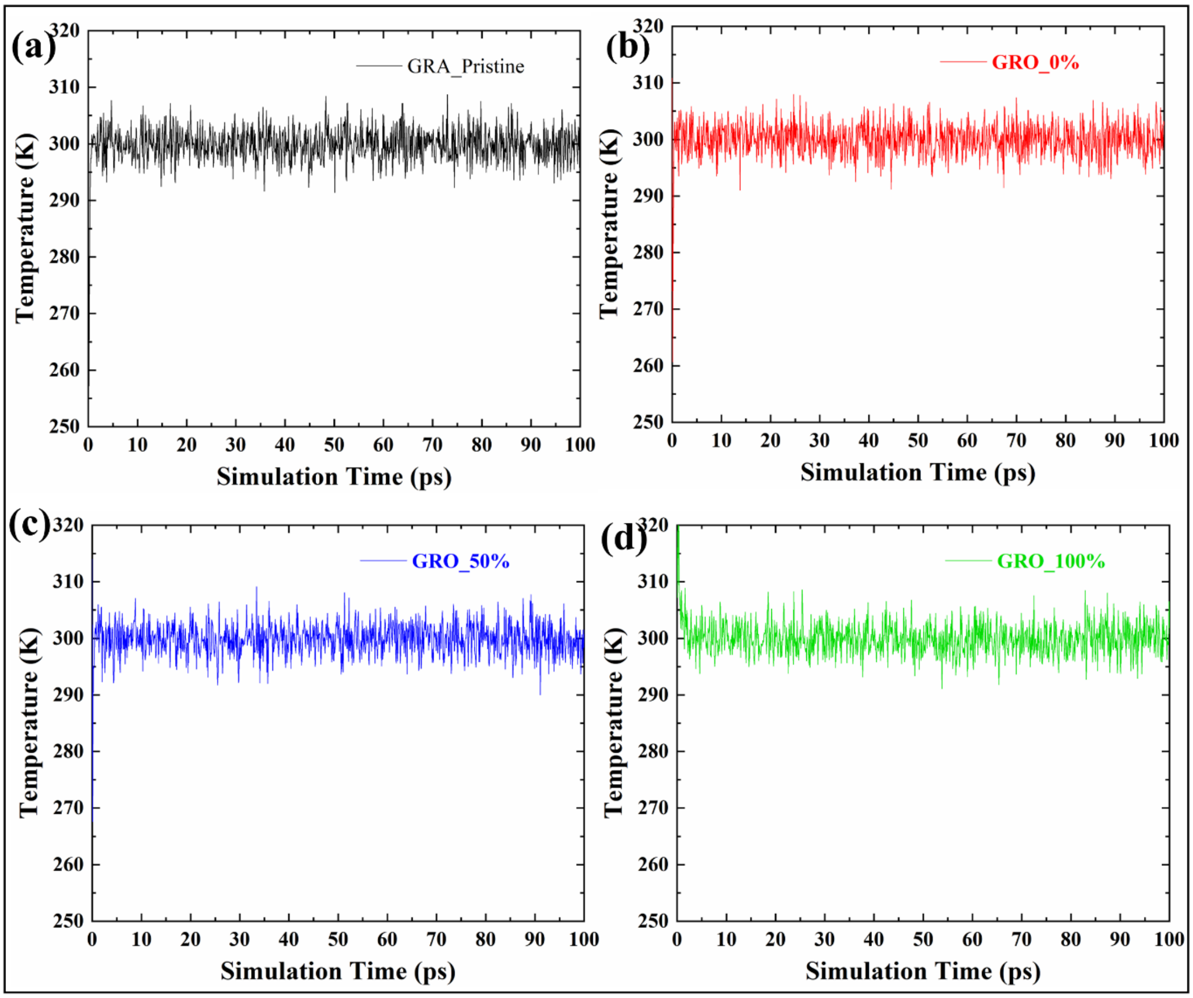

Fig S1. Thermodynamic Equilibration of simulated systems; (a) Pristine Graphene; (b) Graphene oxide with 0% deprotonation; (c) Graphene oxide with 50% deprotonation; (d) Graphene oxide with 100% deprotonation**.**

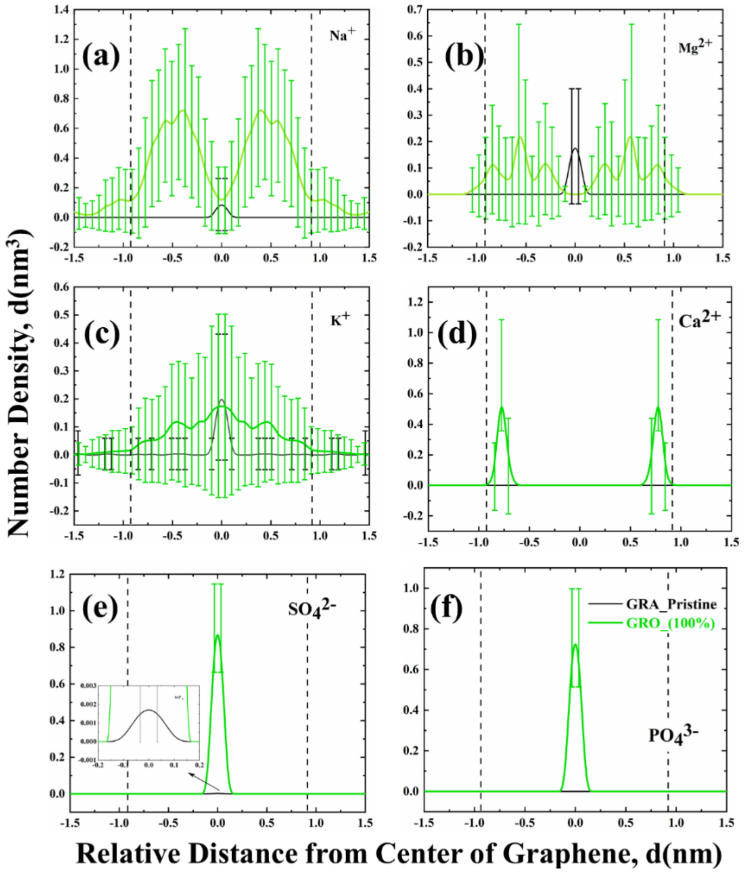

Fig S2. Linear density distribution plots representing the ion condensation at the interface of neutral and charged graphene surfaces with their standard deviation from the mean position. The dotted lines represent the edges of the graphene/graphene oxide sheets in the X-direction. GRA (unfunctionalized graphene), GRO_100% (functionalized graphene) and % (percentage of charge on surface).

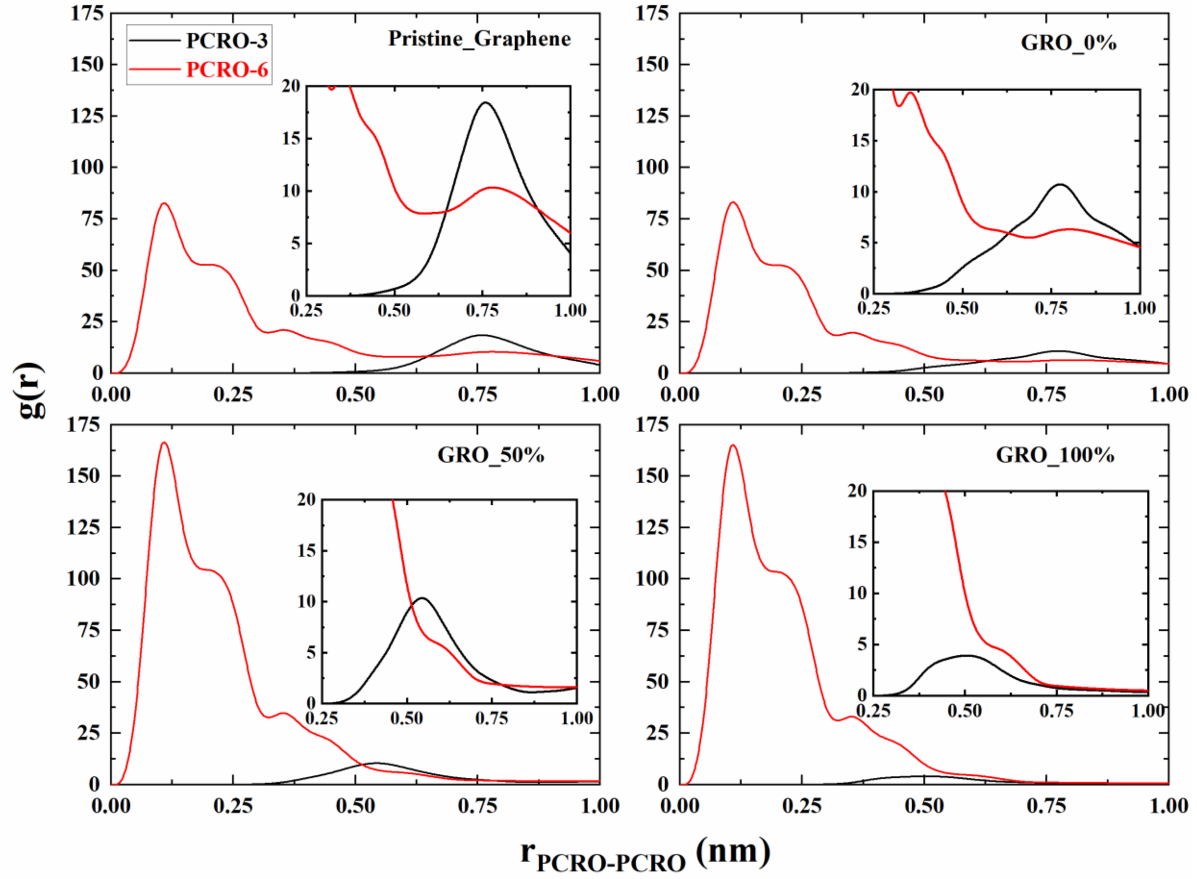

Fig S3. Radial distribution plots depicting the aggregation among the p-Cresol molecules with respect to their concentration in different systems considered in this study
